# RETRACTION: A Meta‐Analysis Examined the Effect of Oxidised Regenerated Cellulose/Collagen Dressing on the Management of Chronic Skin Wounds

**DOI:** 10.1111/iwj.70505

**Published:** 2025-04-21

## Abstract

**RETRACTION:** ChenY. , DuP. , and LvG. , “A Meta‐Analysis Examined the Effect of Oxidised Regenerated Cellulose/Collagen Dressing on the Management of Chronic Skin Wounds,” International Wound Journal 20, no. 5 (2023): 1544–1551, 10.1111/iwj.14009.36480562 PMC10088825

The above article, published online on 08 December 2022, in Wiley Online Library (http://onlinelibrary.wiley.com/), has been retracted by agreement between the journal Editor in Chief, Professor Keith Harding; and John Wiley & Sons Ltd. Following an investigation by the publisher, all parties have concluded that this article was accepted solely on the basis of a compromised peer review process. The editors have therefore decided to retract the article. The authors did not respond to our notice regarding the retraction.

**RETRACTION:** Y. Chen , P. Du , and G. Lv , “A Meta‐Analysis Examined the Effect of Oxidised Regenerated Cellulose/Collagen Dressing on the Management of Chronic Skin Wounds,” International Wound Journal 20, no. 5 (2023): 1544–1551, 10.1111/iwj.14009.36480562 PMC10088825

The above article, published online on 08 December 2022, in Wiley Online Library (http://onlinelibrary.wiley.com/), has been retracted by agreement between the journal Editor in Chief, Professor Keith Harding; and John Wiley & Sons Ltd. Following an investigation by the publisher, all parties have concluded that this article was accepted solely on the basis of a compromised peer review process. The editors have therefore decided to retract the article. The authors did not respond to our notice regarding the retraction.

